# Complete Genome Sequence of Acinetobacter junii Strain INC8271, Isolated from a Patient with Metastatic Cancer and Bacteremia

**DOI:** 10.1128/MRA.00604-21

**Published:** 2021-08-19

**Authors:** Ana Sofía Escobedo-Muñoz, Elena Bello-López, Consuelo Velázquez-Acosta, Patricia Cornejo-Juárez, Patricia Volkow-Fernández, Miguel Ángel Cevallos

**Affiliations:** a Universidad Nacional Autónoma de México, Centro de Ciencias Genómicas, Programa de Genómica Evolutiva, Cuernavaca, México; b Instituto Nacional de Cancerología, Departamento de Infectología, Mexico City, México; University of Maryland School of Medicine

## Abstract

Acinetobacter junii INC8271 was isolated from a cancer patient with polymicrobial bacteremia after biliary stent placement. The complete genome sequence consisted of a chromosome of 3,530,883 bp (GC content, 38.56%) with 3,377 genes, including those encoding 74 tRNAs and 18 rRNAs, and two intact prophage sequences. No antibiotic resistance genes were detected.

## ANNOUNCEMENT

Acinetobacter species are widely distributed in nature. However, some species of this genus, like those grouped within the Acinetobacter baumannii-Acinetobacter calcoaceticus (ABC) complex, are relevant because they are frequently linked with life-threatening infections acquired in the hospital. Affected individuals have multimorbidities and are usually hospitalized in the intensive care unit (1, 2). Some other representatives of the genus, like Acinetobacter junii, are only sporadically associated with human infections (3, 4). Recently, nosocomial infections produced by Acinetobacter spp. not belonging to the ABC complex have increasingly been reported, and some of these isolates already carry genes conferring resistance to antibiotics, including carbapenems (5).

Here, we report the complete genome sequence of Acinetobacter junii INC8271, a strain recovered from a blood sample from a 61-year-old patient with adenocarcinoma of the colon with hepatic metastasis, admitted to a tertiary care hospital in Mexico City. The patient developed polymicrobial bacteremia after an endoscopic retrograde cholangiopancreatography with stent placement. Besides Acinetobacter junii, extended-spectrum beta-lactamase (ESBL) Escherichia coli and pansusceptible Klebsiella pneumoniae were also isolated from the same blood sample. Antibiograms were obtained, using a BD Phoenix system and panel NMIC/ID-406. A. junii INC8271 was susceptible to all antibiotics tested except ampicillin.

For DNA isolation, INC8271 was grown overnight in 5 ml of Luria-Bertani medium at 37°C. Genomic DNA was extracted using a genomic DNA purification kit (Thermo Fisher). Sequencing libraries were prepared at BGI Genomics (Wuhan, China), as described by Huang et al. (6). Briefly, randomly generated DNA fragments (200 to 400 bp) were repaired and 3′ adenylated. The adapters were ligated, and the products were circularized. Single-stranded circular DNAs were formatted as the final library. Paired-end sequencing reads (2 × 150 bp) were obtained using the BGISEQ-2000 platform. Adapter removal and quality trimming of the DNBseq reads were performed using Trim Galore v0.6.4 (https://github.com/FelixKrueger/TrimGalore), yielding 3,992,481 paired-end reads. A long-read library was constructed following the protocol “Genomic DNA by ligation” (SQK-LSK109) (Oxford Nanopore Technologies [ONT]). The DNA was not sheared; it was used directly from purification to library construction. Reads were obtained using the ONT MinION device with the MinION R9.4.1 flow cell. A total of 46,114 reads were obtained with an *N*_50_ read length of 15,467 bp. Base-calling was performed using Guppy software v4.0.14, and adapter sequences were removed using Porechop v0.2.4 (https://github.com/rrwick/Porechop). Hybrid assembly and circularization were performed with trimmed short reads and raw long reads using Unicycler v0.4.7 (7). The assembly statistics were calculated using QUAST v5.0.2 (8). The BUSCO v5.1.2 (9) tool suite was employed to evaluate the completeness of the genome, which was annotated using the Prokaryotic Genome Annotation Pipeline (10). All programs were run with default options.

The genome of INC8271 consists of one circular chromosome of 3,530,883 bp with a GC content of 38.56%, 3,377 genes, 74 tRNAs, 18 rRNAs, 4 noncoding RNAs (ncRNAs), and 3,281 coding DNA sequences (CDS). We were unable to identify any antibiotic resistance genes, including *bla*_OXA_ genes, which are intrinsic to the genomes in many Acinetobacter species, using ResFinder v4.1, RGI v5.2.0, and CARD v3.1.2 (11, 12). Two intact prophage sequences were identified using the PHASTER tool (with lengths of 54.4 kb and 40.9 kb) (13). Two integrative mobile elements were identified using ICEberg v2.0 (14).

### Data availability.

The complete genome sequence of A. junii strain INC8271 has been deposited in GenBank under accession number CP071973.1. The long and short reads used for the genome sequence assembly were deposited in the Sequence Read Archive (SRA) under accession numbers SRR14352129 and SRR14352130.
